# Ethical issues in the export, storage and reuse of human biological samples in biomedical research: perspectives of key stakeholders in Ghana and Kenya

**DOI:** 10.1186/1472-6939-15-76

**Published:** 2014-10-18

**Authors:** Paulina Tindana, Catherine S Molyneux, Susan Bull, Michael Parker

**Affiliations:** Navrongo Health Research Centre, Ghana Health Service, P.O. Box 114, Navrongo, Ghana; KEMRI/Wellcome Trust Research Programme, Kilifi, Kenya; The Ethox Centre, Nuffield Department of Population Health, University of Oxford, Oxford, UK

**Keywords:** Human biological samples, Broad consent, Research collaboration, Sample export, Africa

## Abstract

**Background:**

For many decades, access to human biological samples, such as cells, tissues, organs, blood, and sub-cellular materials such as DNA, for use in biomedical research, has been central in understanding the nature and transmission of diseases across the globe. However, the limitations of current ethical and regulatory frameworks in sub-Saharan Africa to govern the collection, export, storage and reuse of these samples have resulted in inconsistencies in practice and a number of ethical concerns for sample donors, researchers and research ethics committees. This paper examines stakeholders’ perspectives of and responses to the ethical issues arising from these research practices.

**Methods:**

We employed a qualitative strategy of inquiry for this research including in-depth interviews and focus group discussions with key research stakeholders in Kenya (Nairobi and Kilifi), and Ghana (Accra and Navrongo).

**Results:**

The stakeholders interviewed emphasised the compelling scientific importance of sample export, storage and reuse, and acknowledged the existence of some structures governing these research practices, but they also highlighted the pressing need for a number of practical ethical concerns to be addressed in order to ensure high standards of practice and to maintain public confidence in international research collaborations. These concerns relate to obtaining culturally appropriate consent for sample export and reuse, understanding cultural sensitivities around the use of blood samples, facilitating a degree of local control of samples and sustainable scientific capacity building.

**Conclusion:**

Drawing on these findings and existing literature, we argue that the ethical issues arising in practice need to be understood in the context of the interactions between host research institutions and local communities and between collaborating institutions. We propose a set of ‘key points-to-consider’ for research institutions, ethics committees and funding agencies to address these issues.

## Background

For many decades, access to human biological samples, such as cells, tissues, organs, blood, and sub-cellular materials such as DNA, for use in biomedical research, has been central in understanding the nature and transmission of diseases across the globe [1–3]. With the completion of the Human Genome project in 2003 and the advent of new high throughput sequencing technologies, the number of samples that are collected, exported and stored for reuse have increased. For example, in one project, over 100,000 samples have been collected across several developing countries to explore and identify critical mechanisms of protective immunity against malaria [2, 4]. This kind of activity is likely to increase with the recent Human Heredity and Health in Africa Initiative (H3Africa) which seeks to build genomic capacity in Africa [5]. Due to limitations of current scientific infrastructure to process and analyse these samples in many African research institutions, these samples are often exported and sometimes stored in well-established laboratories in high income countries (HICs) [6, 7]. Also, data derived from the analysis of these samples are often shared across research institutions.

The practice of exporting and sharing human biological samples from Africa has led to questions about appropriate mechanisms to safeguard the interests of sample donors; that is research participants and their communities [8, 9]. Specific issues around privacy, benefit-sharing and consent, and particularly the validity of broad consent and what consent should be required for sample export, have been highlighted in the literature [10–14]. For example, Langat’s study of two Kenyan ethics review committees in 2005 revealed that 25 per cent of protocols reviewed stated there was a need for sample storage and reuse, but only half actually informed participants of this at the time of consent [7]. In a survey of participants in a malaria clinical trial in Uganda, also in 2005, Wendler *et al*. found that most participants were willing to permit sample export and storage and waive additional consent for future research, provided the study was approved by an ethics review committee. However, respondents expressed a desire to be informed about the sorts of studies stored samples would be used for [11]. In 2010, a survey of patients in Egypt found that most patients (62%) preferred to have their samples exported to other Arab countries compared to Europe and USA [15]. Recently, two qualitative studies conducted in Nigeria [16] and South Africa [17] reported general community support for the storage and reuse of samples but on condition that the appropriate structures are in place to protect the interests of participants. Overall, the literature suggests that research practices involving human biological samples are increasing; these practices are generally recognised and accepted as essential to advance scientific research; but there are concerns which need to be addressed.

This paper reports on a study which sought to identify the practical ethical issues arising in the collection, export, storage and reuse of human biological samples in the context of international collaborative biomedical research. It is the first study to explore these issues from a diverse range of research actors in sub-Saharan Africa (ethics committee members, researchers, fieldworkers and community members). Specifically, stakeholders’ concerns about these research practices were sought in addition to their views about means of addressing them. Our findings are relevant not only to the countries involved in this research, but also to wider debates in the sub-region.

### Research context and setting

The research was conducted in two research settings in sub-Saharan Africa: Navrongo in Northern Ghana which hosts the Navrongo Health Research Centre (NHRC) and Kilifi in Kenya, which hosts the Kenyan Medical Research Institute (KEMRI)/Wellcome Trust programme (KWTRP). Detailed descriptions of these research institutions have been reported in previous work by Tindana et al. [18]; Molyneux et al. [19]. These two research institutions were purposively selected because they are both involved in major research activities involving international collaborations, and have many research projects involving the collection, storage and in some cases export of human biological samples. They both run a longitudinal health and demographic surveillance system (HDSS) and have had a research relationship with the host community for over twenty-five years, both celebrated their 25^th^ anniversary in 2014. These features provided a platform upon which to address the research questions for this study. The aim of this study was not to compare findings from the KWTRP and NHRC but rather to solicit a broad range of views from various stakeholders across the two settings about these questions.

## Methods

We adopted a qualitative research approach [20–24], including semi-structured interviews and group discussions, to gain an in-depth understanding of the key issues from the perspectives of those who are informed, experienced and affected by sample export, storage and reuse in the context of international research collaborations.

Data collection was carried out in Kenya and Ghana over a period of six months between October 2010 and March 2011. A total of 44 interviews (25 in Kilifi and 19 in Navrongo) and six focus group discussions (3 in Kilifi and 3 in Navrongo) were conducted (Table 1). Interviewees included researchers who design and conduct research; fieldworkers and research assistants who are responsible for collecting samples and data, and for engaging with research participants and their communities (and who often come from local communities); laboratory staff responsible for managing and analysing samples; members of research ethics committees; directors of research institutions; and community representatives. Many interviewees were purposively selected from six projects (three per institution) identified through an audit of all ongoing collaborative research projects involving the collection, long-term storage and export of human biological samples.

**Table 1:** **Sample of stakeholders interviewed**

*Research participants*	*Data collection methods*	*Navrongo/Accra*	*Kilifi/Nairobi*
Researchers/Research assistants	In-depth interviews	12 (10 men and 2 women)	12 (8 men and 4 women)
Cross study key informants (Clinical trials coordinator, lab manager, community liaison, training coordinator)	In-depth interviews and group discussion	1(man)	5 (4 men, 1 woman)
Community facilitators and representatives	Focus group discussions	1 group	1 group (5 men and 1 woman)
Fieldworkers	Focus group discussions	2 groups	2 groups
Ethics committees	In-depth interviews	6 (4 men and 2 women)	8 (5 men and 3 women)
Total data collection activities		22 (19 IDIs and 3 FGDs)	28 (25 IDIs and 3 FGDs)

Themes explored in interviews included rationales for long-term storage and export of samples, perceptions of these practices and of the importance of human tissues to community members, and recommendations for change in guidelines and practice. Issues raised in earlier interviews informed later interviews, and were used as probes to add to our understanding of views. Individual interviews took 45 minutes to 1.25 hours, and group discussions approximately two hours. All interviews were conducted in English except for group discussions with the community representatives in Navrongo which were conducted in the local language (Kasem).

### Data management and analysis

All the audio-recorded individual interviews and focus group discussions were transcribed verbatim by trained transcribers. Two of the interviews were not audio-recorded and were summarized into a table with notes, highlighting the key points raised by the interviewees. Verified transcripts were then imported into Nvivo 8 to facilitate the analysis process.

Data analysis was on-going throughout the study, using a thematic approach [25, 26]. Codes were guided by the objectives of the study and from a close reading of the interview transcripts. Some codes were descriptive such as *types of samples, destination of exported samples, types of study/project, ethics review,* while others were conceptual such as *consent, fairness, trust*, *luck*, *agreements*, *integrity.* Over time, codes were further collated into categories such as *nature of research collaborations, challenges with seeking consent, fairness/justice, governance, capacity building and trust relationships*.

### Ethical considerations

This research was approved by the Oxford Tropical Research Ethics Committee (OXTREC 11.10), the Navrongo Health Research Centre Institutional Review Board (NHRCIRB092) and the KEMRI/National Ethical Review Committee (SCC 1818). Written consent was obtained from all the stakeholders interviewed. All participants were assured of confidentiality.

## Results

There was a general consensus among all stakeholders in this study that there are compelling scientific reasons for the collection, export, storage and reuse of human biological samples for research purposes. There was also general community support for biomedical research. However, a number of practical ethical challenges were identified as important and requiring careful consideration in developing models of good research practice. These include cultural sensitivities around the use of blood samples, concerns with broad consent (defined as consent that allows the use of biological samples and associated data in specific immediate research and future research with ethics approval and for opportunities for withdrawal of consent), given the inevitable uncertainty of future uses, perceptions of unfairness in who gains from sample export and storage, and fears of losing control of samples after export. In the following section, we discuss each of these themes in turn, followed by an overall discussion on the importance of local capacity building and effective research governance.

### Local sensitivities around the use of blood samples

Stakeholders reported that although other human samples such as stool, urine and throat swabs are collected for research purposes, there are particular community apprehensions about the use of blood samples in research. This makes seeking consent for blood sampling a general and major background concern in these research settings; a concern inevitably interwoven with sample export and storage. Apprehensions with blood sampling include possible pain for children, and the potential of the volume of blood taken to cause harm, particularly to sick children. The latter concern is further complicated by the local words for ‘anaemia’, a common problem, directly translating to ‘not enough blood’.

*“I think what the mothers seem to imply is that if you take blood out of my child he/she gets weak. And that makes sense. If you’re cut and you bleed a significant amount you get weak. So for them they can’t correlate how much is too much blood to make my child feel weak. And also it’s distressing to them that their child is being bled” (Kilifi, RES04, Female).*

Interviewees suggested that these worries can be a much greater concern than sample export, and storage:

*“I think in some of the consent forms it is included that the blood may be exported outside for further tests, and usually during consent no issues are raised - it’s only when there are so many blood draws, especially for children, that mothers become apprehensive” (Navrongo: RES08, Female).*

Stakeholders also reported some rumours and apprehensions in the community about researchers ‘selling blood’ in both Kilifi and Navrongo and about ‘devil-worshiping’ specifically in Kilifi. Fieldworkers from and living within local communities attributed these concerns in part to confusion caused by explanations of sample export:

*“There is a whole mix up between misconception and then maybe misinterpretation because when they hear the word “transport”, that notion that all the blood samples are transported separately is not in their mind. They think that all the blood is put together and put in the ship so there is that feeling that if this blood is transported and maybe they think that it is put into buckets, so there is that feeling that they are doing something dubious with this blood because transporting to them and even to me might mean many things packed together to outside countries” (Kilifi: Group Discussions with Fieldworkers-FW 01).*

In both Kilifi and in Navrongo, fieldworkers and community representatives reported that the practice of ‘buying blood’ for blood transfusions in clinical settings has also fuelled rumours about selling of blood. As a community representative explains:

*“When people go to the hospital and they say you are short of blood, they say look for people to give you blood if not buy at the blood bank, so the first idea that came to them was that probably that was [the NHRC’s] way of taking blood from the people to go and sell to patients at the hospital” (Navrongo: Community Representative -03, Male).*

That these perceptions are strongly held is illustrated by several researchers in Kilifi and Navrongo reporting that some mothers of study participants had approached them to ask if blood that had been collected from their children during the course of a research project could be given back to them for transfusion.

In both settings however, it was also recognised that underlying concerns with blood taking include people’s special attachment to blood and (mis)trust:

*“…there’s that connection with the past where people generally were used for other people’s financial gain. It’s all connected to the way people have been treated in the past and that maybe difficult, to disconnect” (Kilifi: RES03, Male).*

*“I think traditionally blood is life and traditionally there are attachments and stories linked to blood. Especially when it comes to research. It goes back to our great grandfathers who attributed blood to the initial white men who used to come and steal blood and just run away so when you talk about blood it triggers many memories and that’s why you have to be very careful” (Kilifi: Group Discussions with Community Facilitators).*

The above concerns contribute to consent for blood sampling often being far from straightforward, and some researchers feeling that they have a responsibility to handle samples that have been entrusted to them appropriately:

*“It's not been easy getting consent to take blood samples from participants and so after explaining deeply about what we are doing, one always has to be careful to protect the blood and to make sure that it's used for what it has been collected for. Participants always have concerns about what the blood will be used for” (Navrongo: RES08, Female).*

To address concerns about blood sampling, export and future uses, stakeholders recommended strengthening understanding of scientific research and of projects that involve human biological samples. Most proposed engaging the general community as well as participants through mechanisms such as incorporating videos into consent processes; videos of what happens to samples and of analytical processes being performed. Most interviewees felt that videos would help potential research participants understand what it means to collect a quantity of blood from a participant and visualize how these samples are actually analysed.

*“…I think we need some kind of audio visual kind of demonstration of the process of taking the sample from the research participant to processing that sample and establishing what really it is we have found in that sample and then how the rest of the sample that we have taken is handled for people to physically and mentally imagine what the samples we take from them go through” (Navrongo: REC 01, Male).*

*Open days* for local residents to visit the institutions’ laboratories were also frequently suggested:

*“We need to go beyond just talking to them but maybe even invite people from some of the communities to come and visit our laboratories. … they should come and see what really happens… come and see how their blood ends up so that it can help to dispel some of the worries and fears that they have. Otherwise those things remain entrenched and it has some implications for the work that we do” (Navrongo: RES03, Male).*

There are on-going community engagement initiatives in both settings, including the above recommended activities in Kilifi. Of interest in Kilifi is that some activities aimed at responding to community suggestions and reducing concerns, has introduced new worries. For example removal from vehicles of the research institutions’ logo (which includes a snake and has reportedly fuelled devil worship rumours), prompted new concerns about what the institution has to hide:

*“…even after the logo had been removed, that created a whole lot of issues surrounding the snake logo” (Kilifi: Group discussions with fieldworkers -FW01).*

These responses suggest that there are limits to how much communication can address some of the more fundamental underlying issues and concerns that were raised above including historical and on-going (mis)trust. Several researchers also hinted that there is a limit to how much sampling for research can be promoted in these settings, and to a responsibility among researchers to minimise volumes of blood requested:

*“I think there’s got to be a balance here, on the one hand we know that to enjoy health and have good medical care…..research has to go ahead to develop tools and this knowledge that is required and for research to go ahead it needs samples. On the other hand I think as researchers we must not therefore use this as a blackmailing tool to the community and demand blood as if we’re vampires (laughs) I think we’ve got to be very sensitive and very judicious as to how much blood we get” (Kilifi: CSKI01, Male).*

### Views on broad consent and future uses of samples

Overall, the difficulty of explaining future uses of samples when these are uncertain at the time of sample collection was widely recognised:

*“I would say the consenting process is difficult because to be able to tell the person who is providing the sample all the possible research questions that it’s going to answer at the time of collection is not possible and also as technology is advancing, even if one had intended to do an analysis using a particular kind of machine or a particular kind of process one maybe able to get more information than originally intended just by using a more sophisticated technology” (Nairobi: REC00, Female).*

Ongoing debates in the literature about the validity of broad consent for future uses of samples were reflected in discussions in this study. Some interviewees argued that since the requirements of full disclosure cannot be assured in broad consent, this cannot be considered as valid consent:

*“This is certainly not valid. What is the consent all about? That you have explained the purpose of the research to the person, the risks and benefits of the research to the individual and s/he has granted consent. So you don’t know the risks involved in using those future samples for any other type of research? How can that be consenting to something that I don’t know. Certainly, people cannot behave that way. We have seen it in a couple of consent forms and this IRB has really been critical about that” (Navrongo: REC03, Male).*

Some researchers noted that whatever is actually said in the consent form, there is a potential for ‘abuse’:

*“…. you cannot rule that out because some scientists may go beyond the boundaries and want to do other things that initially were not disclosed to the study participant. Even though in the consent form, they say these aspects would not be included in whatever analysis they are going to do” (Navrongo: RES013, Male).*

Most stakeholders, especially RECs, fieldworkers and community representatives, recommended a re-examination of current approaches to consent for reuse of samples. The majority view was that it is important to move away from granting *blanket* consent (defined as consent that allows the use of biological samples and associated data for future research of any kind at anytime without restrictions). According to RECs in both Nairobi and Navrongo, there are limits to the acceptability of broad consent. They expect researchers to state in clear terms what stored samples will be used for in future and not to be vague or too general, to avoid possible misuse. One of the implications of this is that it would also require limiting the future use of these samples, for example, to the disease studied during the initial consent:

*“..what we have tended to do is to say future – “related”- research, so if you are collecting the samples for malaria research, that future research should be malaria related, it cannot be HIV or something that has nothing to do with the initial purpose for which the samples were collected” (Navrongo: REC01, Male).*

There is a potential tension between this position from the RECs and that of researchers who highlighted the unknown and open nature of future research, in the context of which these RECs’ expectations and requirements for specifics appear problematic. There was therefore a general appeal from researchers for caution in the implementation of guidelines and policies governing these research practices:

*“If you’re doing research you can’t completely know what the next step is because if you did then you wouldn’t be doing research, you’d just be exploring the known. But if you’re exploring the unknown you can’t anticipate, you can’t anticipate everything you’re going to do” (Kilifi: RES03, Male).*

Most researchers and RECs recommended that the decision to return to participants and their community to re-consent to future uses of samples should be deferred to the local REC. Given that re-consenting individuals may often be difficult, community engagement – specifically consultation with community representatives – was generally seen as one option to decide on whether individual re-consent is necessary in particular situations. This process could also help researchers to identify and address particular local sensitivities with proposed analyses.

*“Re-consenting participants is very difficult but we must find a way of involving the community in the decision making process” (Nairobi: REC09, Male).*

*“…if you use those particular samples for any future research without the knowledge of the community or the donors, if any publication comes out that is adverse, that has consequences for … those particular communities. They could give consent for future use of samples, the community members might not know the implications of that but then when you publish the document and then information is trickling down that oh, you say that in this particular community, women are more likely to deliver kids who end up being prostitutes in future, that community would be stigmatized and then they get to realize that no, you didn’t treat them fairly” (Navrongo: REC03, Male).*

### Researchers’ concerns over sample export: Who gains from research samples?

According to most of the KWTRP and NHRC researchers interviewed, although scientific collaborations are often based on a mutual agreement — the details of which are sometimes spelt out in a material transfer agreement (MTA) — there are often concerns about who will ultimately control the management of transferred samples. Most NHRC researchers raised concerns that local researchers and host institutions would be unable to control how the samples are eventually used once the samples and data leave the local institution:

*“Once the samples get out there, the local collaborators do not really have any control as to who uses it and if it is used and there has to be publications, who should be part of that publication – there should be fairness in the attribution of the contributors to generate knowledge out of that” (Navrongo: RES13, Male).*

Although most researchers indicated that they were aware of what the samples are expected to be used for and how they are to be destroyed and/or used up, some reported that they are unable to account for transferred samples:

*“But it is difficult for me to say I know exactly where these samples are. I know there’s one institution, three institutions, maybe four institutions in the US [involved] but I can’t tell you exactly what is happening. But I know some of the collaborations – but I can’t tell you exactly the outcome of the on-going assays” (Kilifi: RES05, Male).*

There were views that the tendency for there to be a lack of feedback about the fate of exported samples leads to suspicions that samples may be used for other purposes or analysis without the knowledge of the contributing researcher. Furthermore, in the absence of feedback, local researchers might assume that they have samples stored in external laboratories when in reality they have been used up and destroyed after the analysis.

Another concern raised was the lack of recognition for local researchers’ contributions in scientific research collaborations. Many researchers alluded to authorship in scientific publications as a concern and expressed a worry that someone else might end up taking the credit for research results. Some NHRC researchers cited instances where some collaborators had used samples and data without acknowledging the local researchers and the community:

*“Internally I have seen how the samples are handled in other collaborating labs within country where people just pick aliquots of the sample, run their studies, you meet them in international circles, they are making presentations, you see the map of Navrongo and you know that the presenter does not even know Navrongo but you know that this work has come from samples that were taken from a study you were part of, we are not acknowledged, the community, the participants are not acknowledged” (Navrongo: RES05, Male).*

*“.. even when publications came out of that trial, some of us who had worked on that project tirelessly were never included in the publications” (Navrongo: RES15, Male).*

Most researchers suggested that issues such as local control of samples and proper recognition remain pertinent as long as the samples are leaving the immediate environment of the donors and collectors of the samples, even to other parts of the country or within south-south initiatives. This highlights the importance of perceptions of what might perhaps be thought of as ‘moral distance’:

*“.. it doesn’t really mean that the concerns or the issues will be resolved if samples do not go beyond the boundaries of Africa. As long as the sample leaves your immediate lab, I mean the issue of control and access that we are talking about is the same. I don’t think it is easier to go to South Africa than it is to go to New York. I think in many cases it is even easier to go to New York” (Navrongo: REC01, Male).*

*“I am an African, a very proud one but I think .. we must not make the issues African or non-African, we should be a bit more objective than that. If I must handle the samples and test them, I must do so not because I am an African but because I would deliver the expected good results” (Kilifi: CSK01, Male).*

### Research ethics committees’ concerns about the fate of exported samples

Local researchers are not the only stakeholders with concerns about the fate of samples once they have been exported. REC members in Nairobi and Navrongo expressed concerns about their inability to control what happens after granting approval for sample export:

*“…once the samples have left our jurisdiction we really have no control over it. And this is something that really we need to look into, how best can we control the samples that have left here, how best can we ensure that they’re used for the purpose that they were taken and they’re not used for another study” (Nairobi: REC04, Female).*

Currently, there are no mechanisms in place to determine the fate of exported samples. According the stakeholders interviewed, material transfer agreements are not common and until recently were not required by many local RECs. A recent requirement in Kenya is for a local scientist to accompany the samples to the external laboratories as a way of building local personnel capacity and to provide some assurances that the samples are not being used for unapproved research. Although it was not clear if this happens in practice, a REC member in Nairobi explained that this mechanism will also allow for research sustainability at the local level:

*“We feel that for sustainability of research … there should be somebody who would be able to continue spearheading and training others to continue with that intervention and to improve on any intervention that comes as a result of research” (Nairobi: REC00, Female).*

Some researchers questioned the feasibility of this requirement and suggested that projects should be considered on a case-by-case basis. REC members expressed an expectation that although they are unable to monitor the fate of samples overseas, the host institutions could take responsibility for the action of their local researchers and keep the REC updated on the outcome of overseas analysis. This places an additional responsibility on the host institution and local researchers to ensure that their external collaborators are also accountable:

*“But when it leaves our jurisdiction, that is where we have no control over it, extra additional ethical issues arise. we always demand to know where it is going to be stored and who is going to be responsible for it out there. This is all in an effort to protect the participant to ensure that even though the sample has left our custody or our control, we transfer this responsibility to another person and another institution. It’s always our belief that they will comply and use it for that purpose only but again as I said it is something that is based on trust” (Nairobi: REC11, Male).*

Group discussions with the KWTRP community facilitators also highlighted the limitations of RECs to monitor the fate of exported samples:

*“If you talk about exporting samples, so long as it’s being used for the right thing, for the right reason, that is okay but we don’t know, currently we have no structures – even when we talk about ERC, the ERC is there as an academic structure but there are no structures in place to say we are going to monitor to see what happens” (Kilifi: group discussions with community facilitators).*

These responses highlight the local concerns and uncertainties surrounding sample export and the need to identify ways of ensuring that samples that leave the immediate environment of its donors and contributors are used only for agreed and approved research purposes.

### The importance of local capacity building and effective research governance

It is worth noting that none of the stakeholders advocated a *complete* ban on sample collection, export, storage and their reuse and all acknowledged the importance of medical research with an international collaborative dimension. There were also reports of research collaborations that have worked well based on mutual trust, transparency, respect and the scientific leadrship at the institution. Nevertheless, against this background, almost every interviewee expressed the view that it would be desirable to have more effective protective measures and assurances to allay the concerns arising in practice. Potential solutions proposed by interviewees included local capacity strengthening and sustainable research partnerships and relationships.

Regarding local capacity strengthening, most interviewees suggested that international research collaborations should provide more opportunities for strengthening the scientific capacity of host African institutions. These should involve not just the technology and infrastructure needed for the analysis but also training and retaining of local personnel with specialized skills to contribute to the conduct, analysis and publication of research locally. Researchers expressed the view that it is more efficient to conduct good research if the required capacity is available locally:

*“….it’s much easier for us to do good science if we have the capacity to do it here. I think we move forward faster if we have up to date technologies here and staff that are suitably qualified to do it. … we’re also keen to make sure that we’re developing local scientists so it’s not the science of the project that’s governing it, it’s the need for developing a group of scientists for the future of this region” (Kilifi: RES07, Male).*

Specifically highlighted was the need to ensure capacity for analysis locally to minimize sample export:

*“I think that there is a need to rather build capacity here. you know there's too much talk about what happens to samples. I believe all those things will cease to be a problem if the samples were to be analysed here” (Navrongo, RES03, Male).*

Nevertheless, some researchers cautioned that while local capacity strengthening is essential, the nature of some research projects will require the sharing of samples, including overseas sample storage, and thus it is important for all research actors to keep an open mind and not assume that sample export will always lead to exploitation of contributing scientists in Africa.

*“..what is important is the whole research process and researchers’ intentions not to do anything that will be detrimental to individuals and communities” (Kilifi: RES01, Male).*

Most stakeholders also highlighted the centrality of *effective research governance mechanisms*, including the important gate-keeping role of RECs and the need to have clear institutional and national policies and guidelines governing these research practices. Most interviewees suggested that, RECs are the most appropriate system for governing research involving human participants including serving as gate keepers of research samples. Both researchers and REC members suggested that RECs could advise researchers on when communities should be consulted, and if participants should be individually re-contacted for their consent for future research projects:

*“I think that role of export should clearly be defined and supervised by the IRB to make sure that the community’s interest is also preserved” (Navrongo, RES07, Male).*

*“Now the IRBs are there actually to protect the subjects, so I think in certain circumstances they should be able to speak on behalf of the subjects especially if the subject has in the past given them that blanket storage. the IRBs we presume would have been keeping abreast with how things have gone so they should have the power to say that well we think you should be able to go ahead, we are standing in trust for them but at the same time too they should also be able to use their judgment to say that for this particular one we think you still need to go back to the subjects to go and see them, so they should serve as a sieve” (Navrongo, RES17, Male).*

A further point suggested above is for RECs to serve as trustees of research samples. Despite the current challenges with the REC system, many interviewees were of the view that RECs should be given the needed logistic and training support to enable them serve as effective gate-keepers of research samples. It is worth noting that fieldworkers and community representatives in both Kilifi and Navrongo did not specifically mention RECs as a solution spontaneously but only supported the idea of such a system only when they were prompted in the interviews.

## Discussion

In our analysis, we observed that the ethical issues arising from the collection, export and reuse of samples are inter-related. Thus, there is the need to discuss and understand these issues from the perspectives of all research actors and in the context of the research interactions between host research institutions and local communities (what we refer to as micro-level issues) and interactions between collaborating institutions (macro-level issues). Also, we did not find any major differences between the different sexes and age groups in our analysis of the data. The major differences in opinion had more to do with the number of years respondents had been exposed to research and their roles in the research institution.

Our data suggest that biomedical research is evolving rapidly and its success in the future will increasingly depend on access to human biological samples and collaborative partnerships. This is consistent with current literature on biobank research highlighting the important societal value of biobanks in advancing biomedical research [8, 27–30]. It is also suggested that biomedical research is a complex and expensive enterprise requiring the pulling together of expertise and resources to achieve important research goals which include alleviating suffering, advancing knowledge, preserving life and promoting human well-being. Thus, research collaborations will continue to be a key way of moving both the science and ethics of research forward [5, 31, 32]. Our research supports the findings of previous empirical studies in Africa in identifying a high level of general support for biomedical research activities in Africa [17, 33, 34]. However, there is a pressing need for a number of practical ethical concerns to be addressed in order to ensure high standards of practice and maintain public confidence in international research collaborations, particularly those involving the collection, export and reuse of human biological samples.

At the micro-level are local concerns about the use of blood samples in research which have resulted in rumours about too much blood being taken, blood-selling and devil worshipping, as reported in the past in these research settings [19, 35]. Interviewees in this study attributed these concerns in part to the perceived lack of understanding, unfamiliarity, uncertainties and complexities associated with novel research projects such as genetic and genomic research. Thus one proposed solution is to identify innovative and effective ways of communicating the rationale for these scientific research practices to all research actors including fieldworkers, community representatives and research ethics committees to allay these local fears. However, it was also evident that there are deep cultural sensitivities around the use of blood in general which need to be taken seriously. Although we did not seek to compare our findings with non-African perspectives, our data suggests that cultural sensitivities around blood samples are more pronounced in these African settings because of their historical background and cultural attachment to blood. The concerns raised in this study support other claims in the literature linking historical accounts with people’s perception of medical research in general and the local community’s view of some research institutions in Africa as ‘blood-stealing’ organizations [35–37]. As these scholars have also suggested, these cultural sensitivities reveal how local communities are responding to their relationships of dependence and inequality with host research institutions [37]. This raises issues of justice and benefit-sharing as ethical concerns, which require serious attention in determining whether research is ethical or not.

To address concerns about the validity of broad consent, many stakeholders interviewed have proposed the need to identify innovative ways of meaningfully engaging sample donors and their communities, particularly on decisions around the future uses of samples. Clearly, with the growing recognition that research with human biological samples have implications for the wider community and population, most of these suggestions are calls for extending the ethical principle of respect for persons to communities as well. It is worth noting that both KWTRP and NHRC have established mechanisms for communicating with local residents about the institutions’ research activities. The processes involved in these CE strategies have been reported elsewhere [18, 38, 39], and include regular interactions with networks of voluntary community representatives to consult with, public meetings with the general public and community leaders in local villages, community member visits to the research institution, and fieldworker support and training [39, 40]. Interviewees in both sites were confident that these engagement activities are effective ways of improving research literacy and strengthening the trust relationship between the host institutions and the community. In Kilifi for example there are on-going in-depth consultative activities with a diverse range of community members on appropriate forms of benefit-sharing for research, including for studies involving blood sampling [41, 42]. There are also examples of deliberative processes that have proved useful in soliciting public views that could be explored in specific contexts [43, 44]. However, what remains unclear is the extent to which sample donors and communities should be involved in decisions around future uses of samples. Further empirical studies on what methods of community engagement will be most effective in this process would be desirable.

At the macro-level, this research highlights concerns among African researchers and research ethics committees on the fate of exported samples and who stands to gain in research collaborations. These responses also suggest that the issue about local control of research samples and proper recognition does not disappear because the collaboration is just between African institutions. What is important is the nature of the relationship, especially one of trust, that is developed between the various research actors in the collaboration. While some stakeholders have suggested the need for strengthening the capacity of host research institutions to enable much of the research process to be conducted locally and to enhance local control of samples, it was also evident that no level of local capacity can completely eliminate sample export. These include requirements for uniformity of analysis, quality control and the growing demands for sample and data sharing. Researchers’ concerns are also based on principles of justice and calls for some assurance that, irrespective of where research takes place, the interests of the less dominant partners in the collaboration (host African institutions, participants and communities) will be adequately protected, against the background of inevitable sample export. This can also be achieved through clear, transparent and fair research agreements [45, 46] as well as ensuring feedback and accountability on the fate of exported samples. These processes are important to bolster host communities’ confidence in the research enterprise and to protect participants and communities from the effects of misplaced trust.

## Conclusion

Based on our interpretation of this data, we conclude that many of the ethical issues arising in practice cannot be taken in isolation but need to be understood within the context of the interactions between host research institutions and host communities (micro-level) and those between collaborating institutions (macro-level), which in turn are embedded within broader historical and current global contexts of inequity. Secondly, current ethical standards such as requirements for consent and research ethics committees have limitations in responding to the complex, unpredictable and uncertain ethical issues arising from sample storage, export and future research uses. Last but not least, appropriate forms of trust-building and assurances through better consent and community engagement strategies and governance mechanisms are important in moving both the science and ethics of international biomedical research forward and in ensuring the development and implementation of high ethical standards in international collaborative research practice.

## Abbreviations

KWTRP: Kenya Wellcome Trust Research Programme
NHRC: Navrongo Health Research Centre
FGD: Focus group discussions
IDI: Indepth interviews
REC: Research ethics committees
MTA: Material transfer agreements
CE: Community engagement
CR: Community representatives
FW: Fieldworkers
CSKI: Cross study key informant.

## References

[1] Dalal S, Holmes MD, Ramesar RS (2010). Advancing public health genomics in Africa through prospective cohort studies. J Epidemiol Community Health.

[2] MalariaGEN (2008). A global network for investigating the genomic epidemiology of malaria. Nature.

[3] Sirugo G, Hennig BJ, Adeyemo AA, Matimba A, Newport MJ, Ibrahim ME, Ryckman KK, Tacconelli A, Mariani-Costantini R, Novelli G, Soodyall H, Rotimi CN, Ramessar RS, Tishkoff SA, Williams SM (2008). Genetic studies of African populations: an overview on disease susceptibility and response to vaccines and therapeutics. Hum Genet.

[4] Teo Y-Y, Small KS, Kwiatkowski DP (2010). Methodological challenges of genome-wide association analysis in Africa. Nat Rev Genet.

[5] Consortium THA (2014). Research capacity. Enabling the genomic revolution in Africa. Science.

[6] De Vries J, Bull SJ, Doumbo O, Ibrahim M, Mercereau-Puijalon O, Kwiatkowski D, Parker M (2011). Ethical issues in human genomics research in developing countries. BMC Med Ethics.

[7] Langat S (2005). Reuse of samaples: ethical issues encountered by two institutional ethics review committees in Kenya. Bioethics.

[8] Cambon-Thomsen A, Rial-Sebbag E, Knoppers B (2007). Trends in ethical and legal frameworks for the use of human biobanks. Eur Respir J.

[9] Charo R (2006). Body of resea\rch-ownership and use of human tissue. N Engl J Med.

[10] Wendler D, Emanuel E (2002). The debate over research on stored biological samples: what do the sources think?. Arch Intern Med.

[11] Wendler D, Pace C (2005). Research on stored biological samples: the views of Ugandans. IRB.

[12] Hansson MG, Dillner J, Bartram CR, Carlson JA, Helgesson G (2006). Should donors be allowed to give broad consent to future biobank research?. Lancet Oncol.

[13] Weijer C, Miller P (2004). Protecting communities in pharmacogenetic and pharmacogenomic research. Pharmacogenomics J.

[14] Emanuel EJ, Wendler D, Killen J, Grady C (2004). What makes clinical research in developing countries ethical? The benchmarks of ethical research. J Infect Dis.

[15] Abou-Zeid A, Silverman H, Shehata M, Shams M, Elshabrawy M, Hifnawy T, Rahman SA, Galal B, Sleem H, Mikhail N, Moharran N (2010). Collection, storage and use of blood samples for future research: views of Egyptian patients expressed in a cross-sectional survey. J Med Ethics.

[16] Igbe MA, Adebamowo CA (2012). Qualitative study of knowledge and attitudes to biobanking among lay persons in Nigeria. BMC Med Ethics.

[17] Moodley K, Sibanda N, February K, Rossouw T (2014). "It's my blood": ethical complexities in the use, storage and export of biological samples: perspectives from South African research participants. BMC Med Ethics.

[18] Tindana PO, Rozmovits L, Boulanger RF, Bandewar SV, Aborigo RA, Hodgson AV, Kolopack P, Lavery JV (2011). Aligning community engagement with traditional authority structures in global health research: a case study from northern Ghana. Am J Public Health.

[19] Molyneux C, Peshu N, Marsh K (2005). Trust and informed consent: insights from community members on the Kenyan Coast. Soc Sci Med.

[20] Beauchamp TL (1994). Principles and other emerging paradigms in bioethics. Indiana Law J.

[21] Butterfield PS (1989). Qualitative research strategies and methods in health care settings. J Healthc Educ Train.

[22] Mays N, Pope C (2000). Qualitative research in health care. Assessing quality in qualitative research. BMJ.

[23] Ritchie J, Spencer L (1993). Qualitative data analysis for applied policy research.

[24] Denzin NK, Lincoln YS (2000). Handbook of qualitative research.

[25] Boyatzis RE (1998). Transforming qualitative information : thematic analysis and code development.

[26] Fereday J, Mair-Cochrane E (2006). Demonstrating rigor using thematic analysis: a hybrid approach of inductive and deductive coding and theme development. Int J Qual Meth.

[27] Gibbons SM, Kaye J (2007). Governing genetic databases: collection, storage and use. Kings Law J.

[28] Kaye J, Curren L, Anderson N, Edwards K, Fullerton SM, Kanellopoulou N, Lund D, MacArthur DG, Mascalzoni D, Shepherd J, Taylor PL, Terry SF, Winter SF (2012). From patients to partners: participant-centric initiatives in biomedical research. Nat Rev Genet.

[29] Kaye J (2006). Do we need a uniform regulatory system for biobanks across Europe?. Eur J Hum Genet.

[30] Staunton C, Moodley K (2013). Challenges in biobank governance in Sub-Saharan Africa. BMC Med Ethics.

[31] ᅟ (2002). International ethical guidelines for biomedical research involving human subjects. Bull Med Ethics.

[32] Adoga MP, Fatumo SA, Agwale SM (2014). H3Africa: a tipping point for a revolution in bioinformatics, genomics and health research in Africa. Source Code Biol Med.

[33] Van Schalkwyk G, De Vries J, Moodley K (2012). "It's for a good cause, isn't it?" - Exploring views of South African TB research participants on sample storage and re-use. BMC Med Ethics.

[34] Tindana P, Bull S, Amenga-Etego L, De Vries J, Aborigo R, Koram K, Kwiatkowski D, Parker M (2012). Seeking consent to genetic and genomic research in a rural Ghanaian setting: a qualitative study of the MalariaGEN experience. BMC Med Ethics.

[35] Molyneux C, Peshu N, Marsh K (2004). Understanding of informed consent in a low-income setting: three case studies from the Kenyan Coast. Soc Sci Med.

[36] Fairhead J, Leach M, Small M (2006). Where techno-science meets poverty: medical research and the economy of blood in the Gambia, West Africa. Soc Sci Med.

[37] Geissler PW, Pool R (2006). Editorial: Popular concerns about medical research projects in sub-Saharan Africa–a critical voice in debates about medical research ethics. Trop Med Int Health.

[38] Marsh V, Kamuya D, Rowa Y, Gikonyo C, Molyneux S (2008). Beginning community engagement at a busy biomedical research programme: experiences from the KEMRI CGMRC-Wellcome Trust Research Programme, Kilifi, Kenya. Soc Sci Med.

[39] Kamuya DM, Theobald SJ, Munywoki PK, Koech D, Geissler WP, Molyneux SC (2013). Evolving friendships and shifting ethical dilemmas: fieldworkers' experiences in a short term community based study in Kenya. Dev World Bioeth.

[40] Kamuya DM, Marsh V, Kombe FK, Geissler PW, Molyneux SC (2013). Engaging communities to strengthen research ethics in low-income settings: selection and perceptions of members of a network of representatives in coastal Kenya. Dev World Bioeth.

[41] Molyneux S, Mulupi S, Mbaabu L, Marsh V (2012). Benefits and payments for research participants: experiences and views from a research centre on the Kenyan coast. BMC Med Ethics.

[42] Marsh V, Kombe F, Fitzpatrick R, Williams TN, Parker M, Molyneux S (2013). Consulting communities on feedback of genetic findings in international health research: sharing sickle cell disease and carrier information in coastal Kenya. BMC Med Ethics.

[43] Parker M (2005). A deliberative approach to clinical bioethics. Int Lib Ethics Law.

[44] Secko DM, Preto N, Niemeyer S, Burgess MM (2009). Informed consent in biobank research: A deliberative approach to the debate. Soc Sci Med.

[45] Andanda PA (2008). Human-tissue-related inventions: ownership and intellectual property rights in international collaborative research in developing countries. J Med Ethics.

[46] Muula A, M-B JM (2007). Responsibilities and obligations of using human research specimens transported across national boundaries. J Med Ethics.

[CR47] The pre-publication history for this paper can be accessed here: http://www.biomedcentral.com/1472-6939/15/76/prepub

